# Development and optimization of an in-house heterologous ELISA for detection of prednisolone drug in enzyme conjugates using spacers

**DOI:** 10.3389/fimmu.2023.1200328

**Published:** 2023-08-22

**Authors:** Dinesh Kumar, Harinder Singh Oberoi, Harpal Singh, Tulsidas G. Shrivastav, Prudhvi Lal Bhukya, Mansi Kumari, Bidhan Chandra Koner, Subash Chandra Sonkar

**Affiliations:** ^1^ Department of Reproductive Biomedicine, National Institute of Health and Family Welfare (NIHFW), New Delhi, India; ^2^ Quality Assurance Division, Food Safety and Standards Authority of India (FSSAI), New Delhi, India; ^3^ Centre for Biomedical Engineering, Indian Institute of Technology Delhi (IIT-D), New Delhi, India; ^4^ Department of Biomedical Engineering, All India Institute of Medical Sciences Delhi (AIIMS-D), New Delhi, India; ^5^ Rodent Experimentation Facility, Indian Council of Medical Research (ICMR)-National Animal Resource Facility for Biomedical Research (Indian Council of Medical Research (ICMR)-NARFBR), Hyderabad, India; ^6^ Dr. D. Y. Patil Biotechnology and Bioinformatics Institute, Dr. D. Y. Patil Vidyapeeth, Pune, India; ^7^ Multidisciplinary Research Unit, Maulana Azad Medical College and Associated Hospital, New Delhi, India; ^8^ Department of Biochemistry, Maulana Azad Medical College and Associated Hospital, New Delhi, India; ^9^ Delhi School of Public Health, Institute of Eminence, University of Delhi, Delhi, India

**Keywords:** heterologous ELISA, bridge/spacers, prednisolone, antibody, horseradish peroxidase, enzyme conjugates, spacers, immunogens

## Abstract

The introduction of spacers in coating steroid protein complexes and/or enzyme conjugates or immunogens is known to exert an influence on the sensitivity of steroid enzyme immunoassays. We investigated the impact of different homobifunctional spacers, ranging in atomic length from 3 to 10, on the sensitivity and specificity of prednisolone (PSL) enzyme immunoassays. In this study, four homo-bifunctional spacers, namely, carbohydrazide (CH), adipic acid dihydrazide (ADH), ethylene diamine (EDA), and urea (U), were incorporated between PSL and horseradish peroxidase (HRP) for preparing the enzyme conjugate with an aim to improve the sensitivity of the assay without compromising assay specificity. The assays were developed using these enzymes conjugated with antibodies raised against the PSL-21-HS-BSA immunogen. The sensitivity of the PSL assays after insertion of a bridge in the enzyme conjugate was 1.22 ng/mL, 0.59 ng/mL, 0.48 ng/mL, and 0.018 ng/mL with ADH, CH, EDA, and urea as a spacer, respectively. Among the four combinations, the PSL-21-HS-BSA-antibody with PSL-21-HS-U-HRP-enzyme conjugate gave better sensitivity and less cross-reaction. The percent recovery of PSL from the exogenously spiked human serum pools was in the range of 88.32%-102.50%. The intra and inter-assay CV% was< 8.46%. The PSL concentration was estimated in the serum samples of patients on PSL treatment. The serum PSL values obtained by this method correlated well with the commercially available kit (r^2 ^= 0.98). The present study suggests that the nature of the spacer is related to assay sensitivity and not the spacer length.

## Graphical abstract

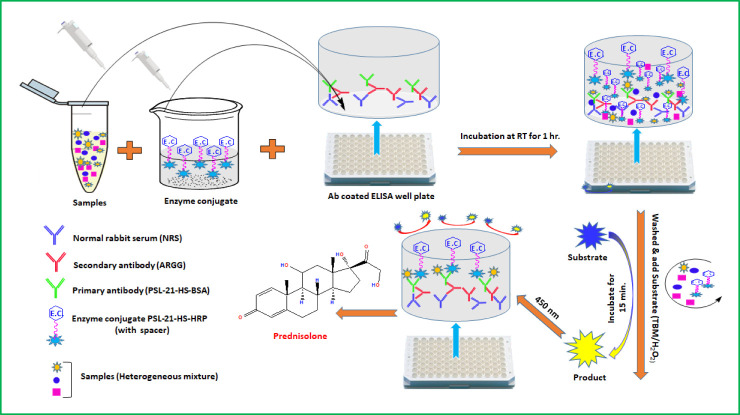

## Highlights

❖ The spacer length is not significant as the spacer structure; it plays a remarkable role in improving the sensitivity of ELISA.
❖ The physicochemical nature of the bridge and steroid is determinant in defining the assay parameters.
❖ The in-house developed assay is simple, sensitive, direct, and convenient to use for prednisolone in serum.
❖ The procedure adopted for coating primary antibodies does not require affinity-purified antibodies.

## 1 Introduction

The PSL (1, 4-Pregnadiene-11β,17α,21-Triol-3, 20-Dione/1-Dehydrocortisol) is a synthetic analog of endogenous cortisol, with potent glucocorticoid and low mineralocorticoid activity, which makes it useful for the treatment and management of a broad range of autoimmune diseases (such as lupus, arthritis, and nephritis). It is also widely used in veterinary medicine, particularly to treat inflammatory diseases, shock, stress, circulatory collapse, and ketonemia, and to lower fever and reduce pain.

There is evidence that long-term exposure to low concentrations of glucocorticoid may increase negative toxic effects on public health, leading to diabetes and metabolic disorders. Prolonged use of therapy doses may cause depression, hypertension, weight loss, muscle atrophy, bone pain, increased susceptibility to infection, sleep disturbances, delayed wound healing, decreased sex hormone production, and a reduction in growth rates in children. Therefore, there is a growing demand for more sensitive, specific, rapid, and cost-effective methods for the determination of the low concentration of PSL residues in the samples.

Compared with physicochemical methods (HPLC, LC-MS/MS, GC-MS, etc.), enzyme-linked immunosorbent assay (ELISA) is rapid, simple, effective, and needs fewer or no sample preparation. ELISAs are presently the most used and successful technique for the immunologically-based detection of a wide variety of antigens. Its popularity has increased rapidly due to its high-throughput competencies, where it is capable of analyzing many samples in a relatively short period of time. It is not always easy to develop a specific and sensitive ELISA for a small molecule such as prednisolone, a synthetic steroid that is not naturally present in the body. One of the most important factors that determine the sensitivity and specificity is the combination of antibody and enzyme conjugate used in the assay (1–10).

The ELISAs for steroids (hapten) developed so far are either in a heterologous or homologous combination. The heterologous or homologous combinations of antibody and enzyme conjugate in steroid enzyme immunoassay (EIA) effects unlabeled steroid recognition which affects the sensitivity of the assay. In heterologous assays, the haptens used as antigens to produce the antibody are different from the haptens used for tagging the enzyme. In homologous immunoassays, the haptens are similar in both cases. It is often found that in a heterologous assay (11–17)certain differences, such as that of site, bridge, or antigen (steroid), exist between the steroid derivatives used for the preparation of the immunogen and enzyme conjugate, while the assay is more sensitive due to improved fitting of the steroid into the antibody binding pocket and reduced bridge recognition (12, 14, 17). However, the homologous combination does not provide satisfactory sensitivity, because the binding affinity of the labeled antigen to the antibody is higher than that of the antigen to be measured (18, 19). Few sensitive homologous assays for steroids have been developed (1–4, 20–29).

A remarkable improvement in the sensitivity of ELISA has been observed by various researchers using spacers between antigens and coupling protein when used as a coating antigen in the antigen immobilized format (14–17) or between antigen and enzyme when used as an enzyme conjugate in the antibody immobilized format (30, 31). It was demonstrated that in the antigen-immobilized format, varying the lengths of the spacer arm of the coating antigen had a significant effect on the sensitivity of ELISA (29, 32–36). A 19-atom linker, an oligoethylene glycol (OEG), was conjugated between progesterone and ovalbumin (OVA) and used as a coating antigen in the surface plasmon resonance (SPR) flow-through biosensor format. Its hydrophilic nature allows good projection of the antigen (progesterone) into the aqueous mobile phase (27–29, 32).

In the antibody-immobilized format, spacers have been coupled between steroid derivative and label protein to minimize the orthodox bridge recognition effects and to overcome steric constraints between the two high molecular weight proteins, i.e., the antibody and label (5–11, 14, 19, 20). Shrivastav et al. studied the differential behavior of spacers such as adipic acid dihydrazide (ADH), ethylenediamine (EDA), carbohydrazide (CH), urea (U), gamma amino butyric acid-ADH (GABA-ADH), and 6-amino caproic acid-ADH (6ACA-ADH) in the steroid-horseradish peroxidase (HRP) enzyme conjugate in the direct ELISA format. The study revealed that the use of a spacer between the steroid and enzyme in the enzyme conjugate increases the sensitivity and specificity of the assay. It has also been reported that the spacer length does not bear any correlation with the assay sensitivity in an ELISA of steroids (1, 5–11, 14, 19, 20). Further, the nature of the spacer (hydrophilic, hydrophobic, flexible, or rigid) is related to assay sensitivity and not to spacer length (5–11, 14, 33). This disparity in the behavior of spacers towards the sensitivity and specificity of assays might be due to the difference in the magnitude of overall forces of attraction between

the antibody and the enzyme conjugates (9). Sathe et al. coupled spacers (urea, ADH and oxy diamine (ODA), bis-oxy diamine (BODA)) between an organophosphorus pesticide and HRP and studied the effect of spacers on the sensitivity of the assay. The use of deliquescent spacers helped to increase the antibody binding signal and improve the sensitivity of the assay (28).

To the best of our knowledge, no reported studies exist on the development of ELISA for PSL using bridge heterology in enzyme conjugates. Also, the PSL derivatives of different bridge lengths are not available commercially to produce enzyme conjugate. It is not easy to synthesize novel hapten derivatives with different bridge lengths as it requires knowledge of synthesis, isolation, and purification of hapten derivatives. To overcome this, we first introduced spacers in the enzyme and purified the spacer-incorporated enzyme using simple dialysis or column chromatography. This spacer incorporated enzyme carboxyl derivative of PSL was coupled. We have incorporated four homobifunctional molecules, ADH (10 atomic length spacer), CH (5 atomic length spacer), EDA (4 atomic length spacer), and U (3 atomic length spacer) as spacers between PSL-21-HS and –NH_2_ blocked HRP for preparation of the enzyme conjugates. The influence of these spacers on assay parameters such as sensitivity, ED_50,_ and specificity has been studied with respect to their length and physiochemical nature.

## 2 Methods

The experimental protocols were approved by the institutional animal ethics committee (IAEC) of the National Institute of Health and Family Welfare (NIHFW), New Delhi, India. All animal experiments were performed in accordance with the guidelines of the Committee for the Purpose of Control and Supervision of Experiments on Animals (CPCSEA), Government of India.

### 2.1 Materials

All solvents, chemicals, and salts used in the present study were of analytical grade and were used without prior purification. All steroids used for the conjugation and cross-reactivity were obtained from Steraloids, Inc. (Newport, RI, USA). Bovine serum albumin (BSA), N-hydroxysuccinimide (NHS), 1-ethyl-3-(3-dimethylaminopropyl) carbodiimide hydrochloride (EDAC.HCl), Freund’s adjuvant complete (FAC), Dioxan, dimethyl formamide (DMF), Tetramethylbenzidine (TMB), Hydrogen peroxide urea (H_2_O_2_.U)/carbamide peroxide, ADH, EDA, CH, urea, and thimerosal were purchased from Sigma Chemical Company (St. Louis, MO, USA). Horseradish peroxidase was purchased from Bangalore Genei, Bangalore, India. An ELISA kit of prednisolone was purchased from Sincere Biotech Co. Ltd., Beijing, China. Microtitre plates were procured from Corning Life Sciences (Tewksbury, MA 01876, USA).

### 2.2 Instrumentation

Tecan Spectra micro-plate reader from Tecan Austria GmbH (5082 Grödig, Austria). A lyophilizer from Haryson (New Delhi, India) and a matrix-assisted laser desorption ionization-time of flight (MALDI-TOF) spectrometer from Bruker Daltonic, Germany.

### 2.3 Preparation of PSL-21-HS-BSA immunogen

**Scheme 1:**
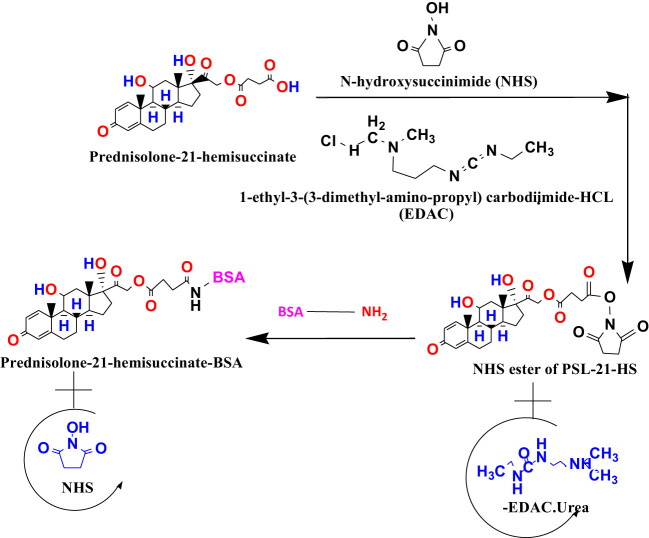
Conjugation of PSL-21-HS (a carboxylic derivative of prednisolone) to BSA.

PSL-21-HS was coupled to BSA by using N-hydroxysuccinimide mediated carbodiimide reaction (21). The plan of the chemical reaction is shown in **Scheme 1**. In brief, 200 μL of both DMF and dioxan, and 100 μL of distilled water containing 10 mg of NHS and 20 mg of EDAC.HCl were added to 5 mg of PSL-21-HS. The reaction mixture was mixed and kept at 4°C for activation overnight. Following the overnight activation, aqueous BSA solution (1 mg/0.5 mL) was added to the activated steroid reaction mixture and vortex-mixed and kept at 4°C overnight for the formation of an amide bond. The PSL-21-HS-BSA conjugate was dialyzed against distilled water for 3-4 changes at 4°C. The dialysate was frozen at -20°C, lyophilized, and stored at 4°C in aliquots of 1 mg for immunization.

### 2.4 Preparation of enzyme conjugate

All the conjugation reactions were carried out as previously described by an active ester method with modification (10).

### 2.5 Preparation of enzyme conjugates with spacer (PSL-21-HS-ADH-HRP, PSL-21-HS-CH-HRP, PSL-21-HS-EDA-HRP, and PSL-21-HS-U-HRP)

**Scheme 2:**
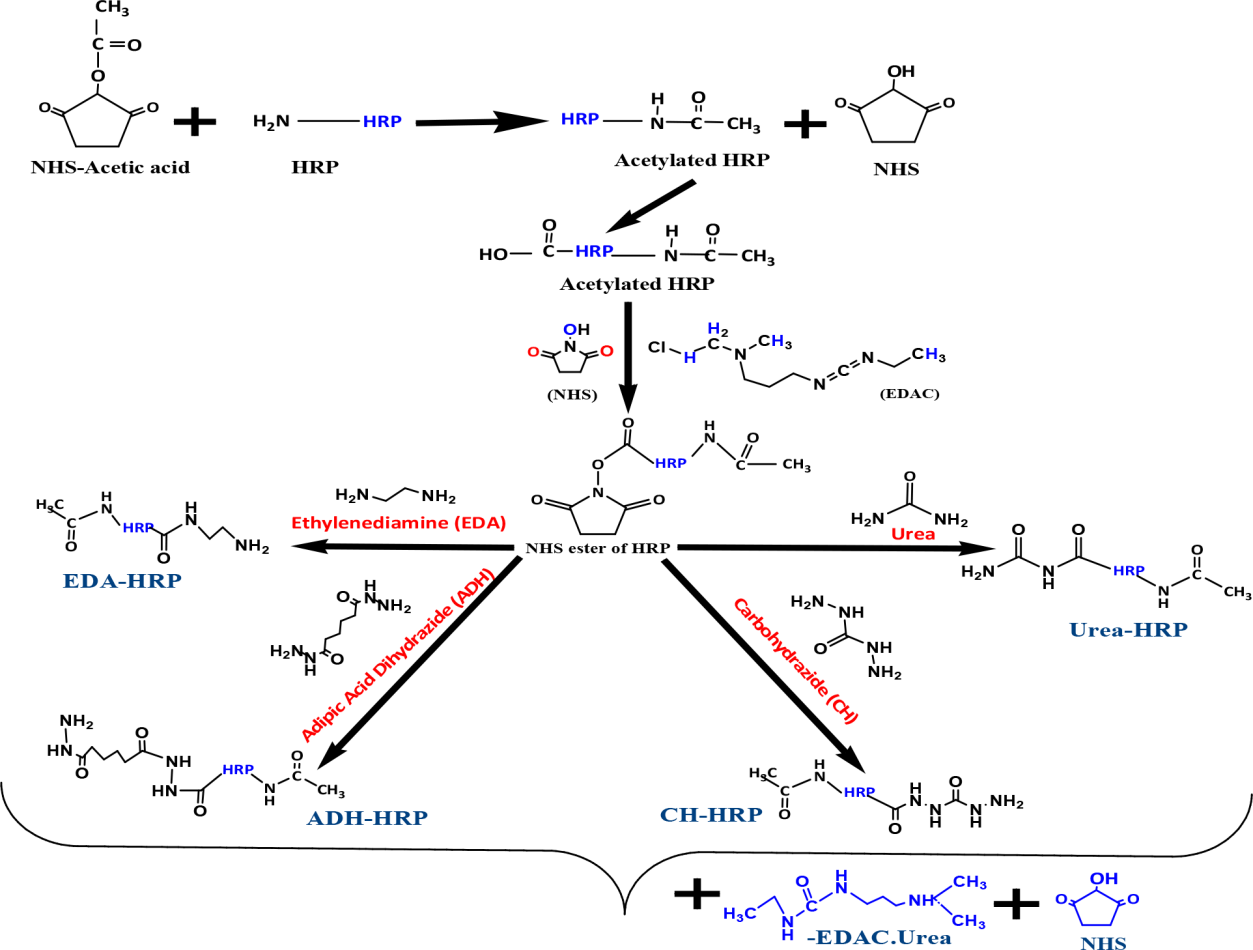
Blocking of the amino group and coupling of spacers (ADH, CH, EDA, and U) to HRP.

**Scheme 3:**
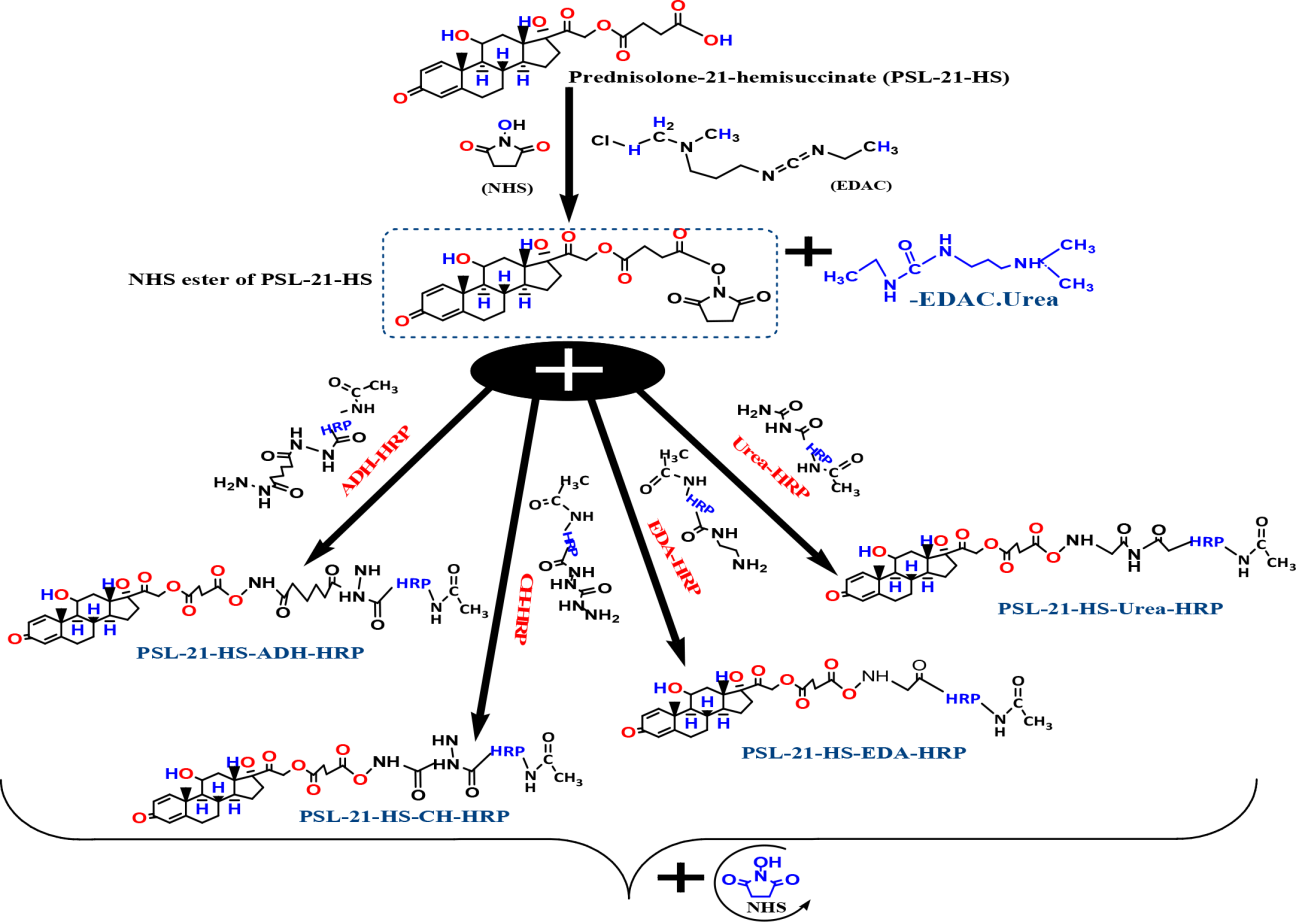
Coupling of prednisolone to spacer-HRP.

The enzyme conjugates with spacers were prepared as shown in **Schemes 2** and **3** by an active ester method (7).

#### 2.5.1 Coupling of spacers to HRP

As per **Scheme 2**, 5 mg of NHS-acetic acid was added to 10 mg of HRP per 1 mL of milli q water. The reaction mixture was vortex-mixed and kept overnight at 4°C to form an amide bond with the –NH_2_ group of HRP that led to the formation of acetylated HRP (blocking of the –NH_2_ group). Thereafter, the reaction mixture was extensively dialyzed against water. Carboxyl moieties of HRP were activated by the addition of 10 mg of NHS plus 20 mg of EDAC.HCl. The activated HRP was then divided into four equal portions; 10 mg each of ADH, CH, EDA, and urea was added to the respective activated HRP portion and kept overnight. After overnight incubation, the reaction mixture was extensively dialyzed against water.

##### 2.5.1.1 Coupling of prednisolone to spacers containing HRP

As per **Scheme 3**, 200 µL each of dioxan and DMF and 100 µL of distilled water containing 10 mg NHS and 20 mg EDAC were added to 10 mg of PSL-21-HS; the reaction mixture was kept at 4°C for activation for 24 hours. The activated PSL-21-HS solution was divided into four equal parts, and each part was added to HRP-U, HRP-EDA, HRP-CH, and ADH-HRP, respectively, and kept for 24 hours at 4°C. Thereafter, each reaction mixture was passed separately through a G-25 column, formerly equilibrated with 10 mM PBS containing 0.01% thimerosal. The brown-colored fractions containing enzyme activity were pooled, and 1% of sucrose, ammonium sulfate, BSA, and an equal volume of ethylene glycol were added to them. The solution was kept at -30°C for future use (10).

### 2.6 Characterization of immunogen and enzyme conjugates by a matrix-assisted laser desorption ionization-time of flight spectrometer

Matrix-assisted laser desorption ionization mass spectrometry (MALDI-MS) has been effectively utilized for the molecular weight determination of various proteins with a claimed limit of 500 000 Da. The practical limit for the routine study of proteins is approximately 300 000 Da with a sensitivity in the low pmol range and an accuracy of up to ± 0.1%. Protein-hapten conjugates were characterized by employing MALDI-TOF. The mass of PSL, BSA, HRP, PSL-BSA, PSL-HRP, PSL-ADH-HRP, PSL-CH-HRP, PSL-EDA-HRP, and PSL-U-HRP were determined using a MALDI-TOF spectrometer. The 1 mg of sample/mL of distilled water was mixed with 1 mL of a matrix (3, 5-dimethoxy-4-hydroxycinnamic acid (Sinapinic acid) was prepared in 33% acetonitrile) in a 1:1 ratio (v/v), thereafter, 0.5 µL of 0.1% trifluoroacetic acid (TFA) was added. The samples were analyzed in linear mode with a 337 nm laser light at 30 kV and spectra for each sample were recorded at a threshold laser irradiance for 50 shots and the data obtained were analyzed using the software provided with the system (22, 30). Each spectrum contained at least two larger and shorter peaks and were in singly (M+H) ^+^ and doubly (M+2H)^2+^ charged states. The molecular weight of each sample was calculated from the singly centroid (M+H) ^+^ peak. The number of haptens attached per molecule of protein was determined using the formula:


$$\text{Number of haptens }=\;\frac{\left[\text{conjugate molecular weight }\left(\text{MW}\right)\right]-\left[\text{Protein }\left(\text{BSA}/\text{HRP}\right)\text{ MW}\right]}{\left(\text{hapten }\left(\text{PSL}\right)+\text{spacer MW}\right)}$$

### 2.7 Immunization of rabbits and collection of antiserum

The polyclonal antibody was raised in New Zealand white rabbits against PSL-21-HS-BSA as per the procedure described elsewhere (20). Briefly, PSL-21-HS-BSA (1mg) was dissolved in 0.9% saline (0.5 mL) and emulsified with 0.5 mL of Freund’s complete adjuvant. The prepared emulsion (250 μL) was injected intramuscularly in each limb of the rabbits. Primarily, the first five booster injections were prepared with Freund’s complete adjuvant and given per week and were further followed by monthly booster injections prepared using Freund’s incomplete adjuvant. Further, the rabbits were bled after 9-14 days after the booster dose injections. Blood was collected from the rabbits and after clotting, antiserum was separated by centrifugation at 1000 g for 15 min. The separated antiserum was collected and stored at -30°C.

### 2.8 Collection of normal rabbit serum and generation of anti-rabbit anti-serum in goat

For the collection of normal rabbit serum, blood was withdrawn from the non-immunized New Zealand white rabbits. After clotting, the blood was centrifuged at 2000 rpm for 10 min. The separated serum was collected and stored as NRS at -30°C until use. The anti-rabbit anti-serum was generated in goats (ARASG) by immunizing the immunoglobulin (IgG) of rabbits (1–3, 21). Further, the booster injection blood was collected from the goat and centrifuged after clotting. The obtained antiserum was collected and stored at -30°C for further use.

### 2.9 Coating of antibody to microtiter plates

The 96-well microtitre plates were coated using the immunobridge technique for primary antibody immobilization (11). Briefly, 250 µL of normal rabbit serum (NRS) diluted (1: 250) in water was dispensed into each well and incubated overnight at 37°C. Following incubation, the plate was washed 2-3 times using a washing solution (buffer E). To the NRS coated wells, 250 µL of 1:1000 diluted anti-rabbit anti-serums in goat (ARASG) was added and incubated for 5 hours at 37°C or overnight at 4°C. Thereafter the plate was washed carefully using a washing solution (buffer E). The raised antiserum against immunogen (PSL-21-HS-BSA) was serially diluted in antibody dilution buffer to 1:500, 1:1000, 1:2000, and 1:4000, and 150 μL was added per ARASG coated wells (single dilution per eight-well strip). For non-specific binding (NSB), 150 μL of antibody dilution buffer was added in a separate ARASG-coated eight-well strip and incubated for 2 hours at 37°C. The unabsorbed antibody was then decanted and the microtitre plate was washed with buffer E. Thereafter, 250 μL of buffer ‘D’ was added and kept at room temperature (RT) for 1 hour to block the unoccupied sites of the plate. The contents of the plate were decanted, packed, and stored at 4°C in zip-lock bags for further use and the plate was dried at RT.

#### 2.9.1 Determination of optimal dilution of primary antibody and enzyme conjugates

To determine the amount of immobilized primary antibody and enzyme conjugates required to develop the assay, 100 µL of serially diluted (1:500, 1:1000, 1:2000, and 1:4000) enzyme conjugate in buffer B (PSL-21-HS-ADH-HRP, PSL-21-HS-CH-HRP, PSL-21-HS-EDA-HRP, or PSL-21-HS-U-HRP) was added in the above coated plates for respective assays (one dilution per two wells in vertical fashion). The plates were incubated at RT for 1 hour. Unbound contents were decanted and the plate was washed with buffer E. The bound enzyme activity was assessed by adding 100 µL of TMB/H_2_O_2_ substrate to each well followed by incubation at RT for 15 min. The reaction was stopped by the addition of 100 μL of 1 N HCl, and the color intensity was measured at 450 nm in a Tecan-spectra ELISA plate reader. The dilutions of antiserum and enzyme conjugate that showed maximum zero binding (optical density more than 2.5) and minimum nonspecific binding (optical density less than 0.1) were selected for each combination for assay development.

#### 2.9.2 Preparation of prednisolone standard

Eight PSL working standards (0.0, 0.62, 1.25, 2.5, 5.0, 10.0, 20.0, and 40.0 ng/mL) were prepared in stripped pooled serum. The stripping of steroids from the serum was carried out by adding activated charcoal at a concentration of 50 mg/mL to pooled serum, stirring for two hours at 45 °C, and centrifuging at 3000 x g to eliminate the charcoal, followed by filtration through 0.45 μM membrane filter. Thimerosal 0.01% was added as a preservative.

#### 2.9.3 Preparation of recovery pools

Six recovery pools were prepared in the serum by spiking the serum with different known concentrations of prednisolone, viz., 0.0, 0.62, 2.5, 5.0, 15.0, and 25.0 ng/mL.

#### 2.9.4 Standard displacement assay (assay procedure)

To the antibody-coated wells (PSL-21-HS-BSA-antibody), 100 µL of different concentrations of the standard were added in duplicate. Next, 100 µL of the working dilution of the enzyme conjugates (PSL-21-HS-ADH-HRP, PSL-21-HS-CH-HRP, PSL-21-HS-EDA-HRP, or PSL-21-HS-U-HRP) was added to all of the wells. The incubation, washing, and measurement of the bound enzyme activity were as described in section 2.9.2.

#### 2.9.5 Measurement of serum prednisolone by commercial ELISA kit

Five working standards (0, 15, 30, 60, 120, and 240 pg/mL) were freshly prepared by serially diluting the stock standard with diluent. To the pre-coated wells, 10 µL of different concentrations of standards or samples were added followed by 40 μL of sample diluent and incubated for 30 min at 37 °C. Thereafter, the plate was washed thoroughly using washing solution and 50 μL of HRP-conjugate was added to all the wells except the blank well and incubated at 37°C for 30 min. The wells were washed again with a washing solution, and the bound enzyme activity was developed by adding 100 µL of TMB/H_2_O_2_ substrate to each well and incubating at 37°C for 15 min. The enzymatic reaction was stopped by adding 100 μL of stop solution, and the color intensity was measured at 450 nm in a Tecan-spectra ELISA plate reader (TECAN, Austria).

## 3 Data analysis

### 3.1 Preparation of the standard curve, determination of affinity constant, and sensitivity

The standard curve was plotted using GraphPad Prism 6.0 and Microsoft Excel software. The concentration was plotted on the X-axis (log scale) and the A/A0x100 on the Y-axis. The values of the unknown samples were calculated by an in-house–developed personal computer program written in QBASIC language using the logit–log linear regression method according to the method described elsewhere (5). The affinity constant of the PSL antibody for PSL was estimated by a Scatchard plot according to the method described elsewhere (10). The sensitivity of the developed method was determined according to the method described elsewhere (21, 32–41).

### 3.2 Goodness of fit

The goodness of fit was expressed as the estimated non-linear squared correlation coefficient (R^2^) of the standard data. An R^2^ value that approaches 1.0 is indicative of a precise fit for the data to the standard curve.

## 4 Results

### 4.1 Determination of hapten density by MALDI-TOF spectrometry

#### 4.1.1 In immunogen

**Figure 1:**
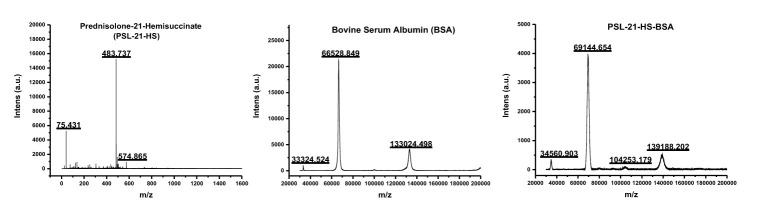
Mass spectra of PSL-21-HS, BSA, and PSL-21-HS-BSA by MALDI-TOF.

The molecular weights of PSL-21-HS, BSA, and PSL-21-HS-BSA were determined by MALDI-TOF and found to be 483.737 Kelo Dalton (KDa), 69144.654 KDa and 66528.848 KDa respectively as shown in **Figure 1**. The quantity of hapten (PSL) density was calculated by the formula given under section 2.6 and found to be 5.40 per molecule of BSA as shown in **Table 1**.

**Table 1:** Estimation of hapten density in immunogen.

Sample	Analyte MW (KDa)	Conjugate MW (KDa)	Change in mass (Conjugate MW – BSA MW)	Hapten density (Change in mass/hapten M.W.)
Prednisolone (PSL)-21-HS	483.737		-	-
Bovine Serum Albumin (BSA)	66528.848		–	–
PSL-21-HS-BSA	-	69144.654	2615.806	5.40

#### 4.1.2 In enzyme conjugates

**Figure 2:**
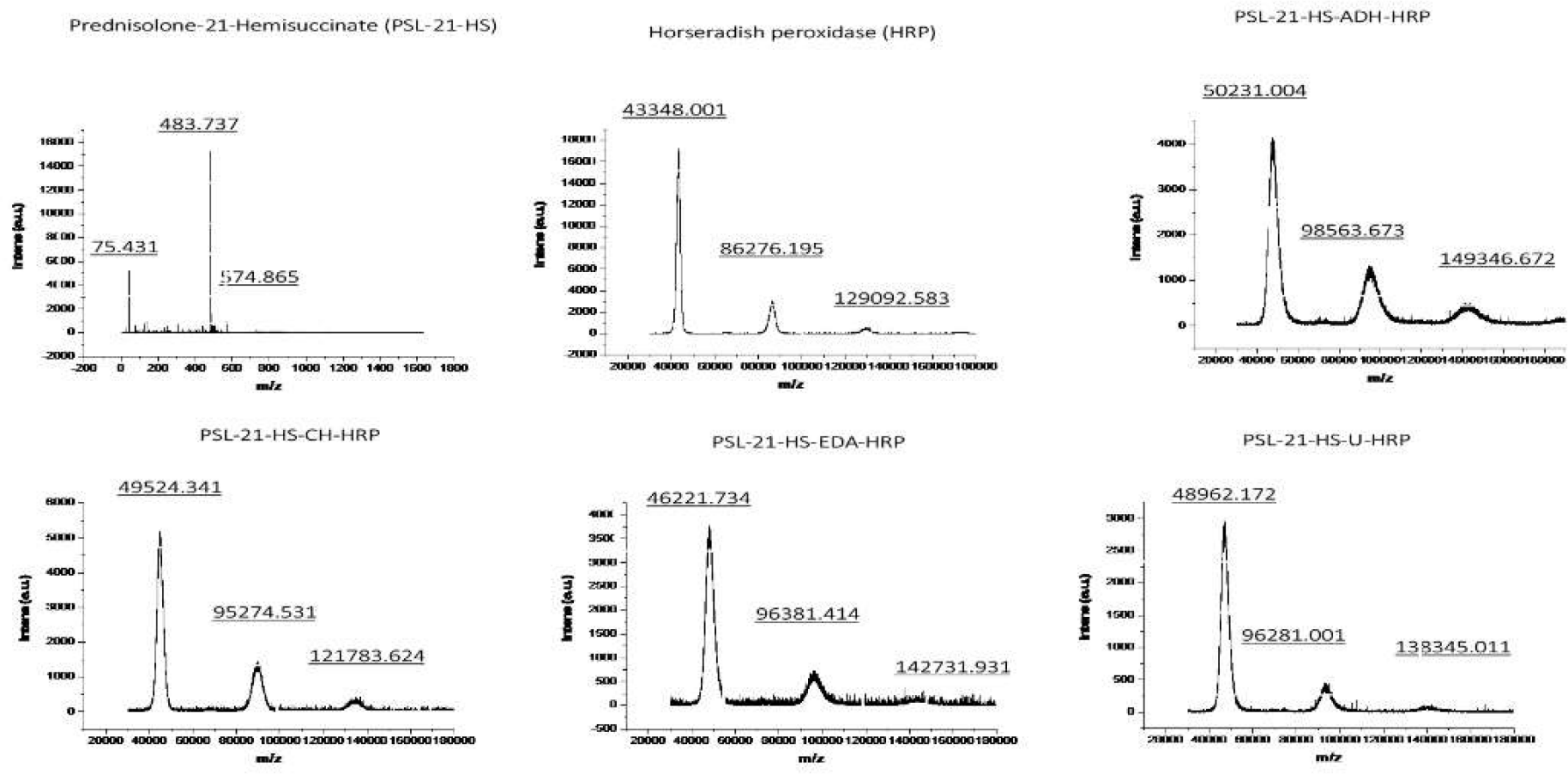
Mass spectra of PSL-21-HS, HRP and enzyme conjugate with spacers by MALDI-TOF.

The molecular weights of HRP, PSL-21-HS, PSL-21-HS-ADH-HRP, PSL-21-HS-CH-HRP, PSL-21-HS-EDA-HRP, and PSL-21-HS-U-HRP were determined by MALDI-TOF and found to be 43348.001 KDa, 483.737 KDa, 50231.004 KDa, 49524.341 KDa, 46221.734 KDa, and 48962.172 KDa, respectively as shown in **Figure 2**. The quantity of hapten (PSL) density was calculated by the formula given in section 2.6 and found to be 10.46, 10.76, 5.28, and 10.32 per molecule of the PSL-21-HS-ADH-HRP, PSL-21-HS-CH-HRP, PSL-21-HS-EDA-HRP, and PSL-21-HS-U-HRP enzyme conjugates respectively. The observed mass, changes in mass, and the hapten density of enzyme conjugates are shown in **Table 2**.

**Table 2:** Estimation of hapten density in enzyme conjugate.

Sample	Analyte MW	Conjugate MW(KDa)	Change in mass (conjugate MW – HRP MW)	Hapten density (change in mass/hapten+spacer M.W.)
Horseradish peroxidase (HRP)	43348.001 KDa			
Prednisolone-21-hemisuccinate (PSL-21-HS)	483.737 KDa			
Adipic acid dihydrazide (ADH)	174.2 g/mol			
PSL-21-HS-ADH-HRP	–	50231.004	6883.00	10.46
Carbohydrazide (CH)	90.09 g/mol			
PSL-21-HS-CH-HRP	–	49524.341	6176.34	10.76
Ethylenediamine (EDA)	60.10 g/mol			
PSL-21-HS-EDA-HRP	–	46221.734	2873.733	5.28
Urea (U)	60.06 g/mol			
PSL-21-HS-U-HRP	–	48962.172	5614.171	10.32

### 4.2 Dose-response study

**Figure 3:**
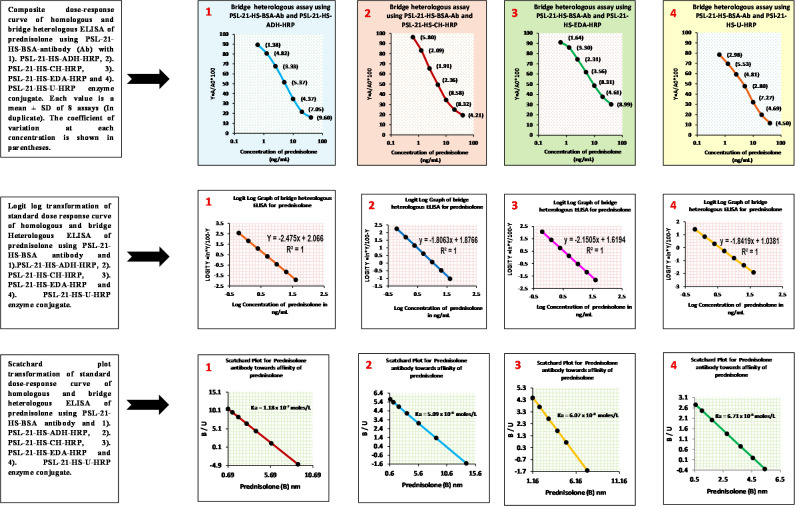
Composite dose-response, logit-log, and Scatchard plot curves of the homologous and bridge heterologous ELISA of prednisolone using PSL-21-HS-BSA antibody PSL-21-HS-HRP with (ADH, CH, EDA, and U) different length spacer–containing enzyme conjugates.

The dose-response studies of the four enzyme conjugates PSL-21-HS-ADH-HRP, PSL-21-HS-CH-HRP, PSL-21-HS-EDA-HRP, and PSL-21-HS-U-HRP were carried out with the antibody raised against PSL-21-HS-BSA. **Figure 3** depicts the composite standard curves of the ELISA for prednisolone using the PSL-21-HS-BSA antibody with PSL-21-HS-HRP (with different length spacer) enzyme conjugates, where concentrations of PSL are plotted on the X axis and bound fraction (A/A0%) on the Y axis. The CVs for the A/A0 ratio of each standard ranged from 1.38% to 9.93% for the enzyme immunosorbent assays. Each value is a mean ± SD of eight assays (in duplicate). Thus, the standard curves achieved over several assays remained stable and precise. The slope and intercept of the curves were calculated by logit–log transformation of standard curve data, the affinity constant of the PSL antibody for the PSL was estimated by a Scatchard plot, and the goodness of fit was the estimated non-linear squared correlation coefficient (R^2^) of the standard data and is shown in **Table 3**.

**Table 3:** Slope (m), intercept (c), sensitivity, affinity, ED_50_, and R² of prednisolone assays, using PSL-21-HS-BSA-antibody and PSL-21-HS-HRP (with spacers) enzyme conjugates.

Assay combination of PSL-21-HS-BSA- antibody with	Slope (m) and intercept (c)		Sensitivity of the assay (ng/mL)	Affinity of the assay (mole/L)	ED_50_ (ng/mL)	R²
	m	c				
PSL-21-HS-ADH-HRP Enzyme conjugate	-2.47	2.06	1.22	1.18 x 10^-7^	6.79	0.9
PSL-21-HS-CH-HRP Enzyme conjugate	-1.80	1.87	0.59	5.07 x 10^-8^	4.03	1.0
PSL-21-HS-EDA-HRP Enzyme conjugate	-2.15	1.61	0.48	4.71 x 10^-8^	3.60	1.0
PSL-21-HS-U-HRP Enzyme conjugate	-1.84	1.03	0.018	5.09 x 10^-8^	1.89	1.0

### 4.3 Sensitivity

The lower detection limit of the PSL assay after insertion of the spacers in the enzyme conjugate was 1.22 ng/mL (ADH spacer), 0.59 ng/mL (CH spacer), 0.48 ng/mL (EDA spacer), and 0.018 ng/mL (urea spacer) and are shown in **Table 3**. Among the four combinations, the PSL-21-HS-BSA antibody and PSL-21-HS-U-HRP enzyme conjugate had better sensitivity.

### 4.4 Specificity

The specificity of the anti-PSL-21-HS-BSA-antiserum with PSL-21-HS-U-HRP, PSL-21-HS-EDA-HRP, PSL-21-HS-CH-HRP, and PSL-21-HS-ADH-HRP was estimated as the percentage of cross-reaction with commercially available 53 analogous steroids. Only 4 to 5 steroids showed cross-reaction and are shown in **Table 4**. Among the four combinations, the PSL-21-HS-BSA-antibody with the PSL-21-HS-U-HRP-enzyme conjugate had better sensitivity and less cross-reaction. The percentage of cross-reaction was calculated from the following formula:

**Table 4:** Cross-reactivity of analogous steroids in bridge heterologous ELISA of PSL using the PSL-21-HS-BSA antibody and PSL-21-HS-HRP (with spacers) enzymes conjugate.

Steroid Measured	% Cross-reactions with PSL-21-HS-EDA-HRP	% Cross-reactions with PSL -21-HS-Urea-HRP	% Cross-reactions with PSL-21-HS-CH-HRP	% Cross-reactions with PSL-21-HS-ADH-HRP
C-27 Steroid				
Cholesterol	<0.025	<0.025	<0.025	<0.025
C-22 Steroid				
Danazol	<0.025	<0.025	<0.025	<0.025
C-21 Steroid				
Progesterone	4.7	1.65	2.21	9.46
5 α Dehydro Progesterone	<0.025	<0.025	<0.025	<0.025
5 β Dehydro Progesterone	<0.025	<0.025	<0.025	<0.025
17α 20β Dioh Progesterone	<0.025	<0.025	<0.025	<0.025
17α 20 α Dioh Progesterone	<0.025	<0.025	<0.025	<0.025
11α OH Progesterone	<0.025	<0.025	<0.025	<0.025
16-dehydroprogesterone	<0.025	<0.025	<0.025	<0.025
Medroxy Progesterone Acetate (MPA)	<0.025	<0.025	<0.025	<0.025
Pregnenolone	<0.025	<0.025	<0.025	<0.025
17α-OH progesterone	21.25	6	12.5	14
17α-OH pregnenolone	<0.025	<0.025	<0.025	<0.025
5 Pregnene 3 β 20 α diOL	<0.025	<0.025	<0.025	<0.025
5α Pregnane 3β, 20α diOL	<0.025	<0.025	<0.025	<0.025
5α Dihydropregnanolone	<0.025	<0.025	<0.025	<0.025
5β-pregnane-3,20-dione	<0.025	<0.025	<0.025	<0.025
Betamethosone	<0.025	<0.025	<0.025	<0.025
Pregnanediol	<0.025	<0.025	<0.025	<0.025
Cortisol	11.6	4.5	12.5	10.4
5 α Dihydro Cortisol	<0.025	<0.025	<0.025	<0.025
5 β Dihydro Cortisol	<0.025	<0.025	<0.025	<0.025
Prednisolone	100	100	100	100
Prednisone	42	8	27	24
Aldosterone	<0.025	<0.025	<0.025	<0.025
Dexamethasone	<0.025	<0.025	<0.025	<0.025
Flutamide	<0.025	<0.025	<0.025	<0.025
Corticosterone	8.6	<0.025	7.48	4.44
5 α Dihydro Corticosterone	<0.025	<0.025	<0.025	<0.025
5 β Dihydro Corticosterone	<0.025	<0.025	<0.025	<0.025
Cortisone	<0.025	<0.025	<0.025	<0.025
5 α Dihydro Cortisone	<0.025	<0.025	<0.025	<0.025
5 β Dihydro Cortisone	<0.025	<0.025	<0.025	<0.025
Deoxycorticosterone (DOC)	<0.025	<0.025	<0.025	<0.025
C-19 Steroid				
Testosterone	<0.025	<0.025	<0.025	<0.025
6 Dehydro Testosterone	<0.025	<0.025	<0.025	<0.025
17 α Methyl Testosterone	<0.025	<0.025	<0.025	<0.025
11 Keto testosterone	<0.025	<0.025	<0.025	<0.025
Dihydrotestosterone (DHT)	<0.025	<0.025	<0.025	<0.025
Etiocholanolone	<0.025	<0.025	<0.025	<0.025
Dehydroepiandrosterone (DHEA)	<0.025	<0.025	<0.025	<0.025
Dehydroepiandrosterone Sulfate (DHEAS)	<0.025	<0.025	<0.025	<0.025
Dehydroisoandrosterone	<0.025	<0.025	<0.025	<0.025
Androstenedione	<0.025	<0.025	<0.025	<0.025
Androstanedione	<0.025	<0.025	<0.025	<0.025
Androsten 3 β, 17 β -Diol	<0.025	<0.025	<0.025	<0.025
Androstenediol	<0.025	<0.025	<0.025	<0.025
Mesterolone	<0.025	<0.025	<0.025	<0.025
Nandrolone	<0.025	<0.025	<0.025	<0.025
C-18 Steroid				
Estrone	<0.025	<0.025	<0.025	<0.025
Estrone 3-Glu (E_1_ 3G)	<0.025	<0.025	<0.025	<0.025
Estradiol	<0.025	<0.025	<0.025	<0.025
Estriol	<0.025	<0.025	<0.025	<0.025
2 Methoxy Estradiol	<0.025	<0.025	<0.025	<0.025


$$\text{cross reaction }=\;\frac{\text{concentration of prednisolone},\text{ required for }50\text{\% inhibition}}{\text{concentration of related steroid},\text{ required for }50\text{\% inhibition }}\times 100$$

### 4.5 Selection of the best combination of antibody and enzyme conjugate

Since, among all four combinations, anti-PSL-21-HS-BSA and PSL-21-HS-U-HRP enzyme conjugate had better sensitivity, ED_50_, affinity, and specificity thus, this combination was further studied for analytical variables such as recovery, precision, and correlation coefficient.

### 4.6 Recovery

The ability of an assay to accurately quantify PSL in serum was tested. **Table 5** represents the percentage of recoveries of known amounts of PSL added to five aliquots of serum pools. After spiking, the concentration of PSL was measured and recovery was intended for each pool. The recovery ranged from 88.32 – 102.50%.

**Table 5:** Recovery of prednisolone from exogenously spiked human serum pools using the PSL-21-HS-BSA-antibody and PSL-21-HS-Urea-HRP-enzyme conjugate.

Human serum pools	Prednisolone added (ng/mL)	Prednisolone observed (ng/mL)	Prednisolone expected (ng/mL)	% Recovery
Pool A (Basal)	–	0.75	––	–
Pool B	0.62	1.21	1.37	88.32
Pool C	1.25	2.05	2.00	102.50
Pool D	5.0	5.55	5.75	98.26
Pool E	15.0	`15.50	15.75	98.41
Pool F	25.0	25.80	25.75	100.19

The percentage of recovery was calculated by using the formula: (C - B)/A * 100. Where A = a known amount of analyte, B = a base (B), and C = measuring the concentration.

### 4.7 Precision

The levels of precision are estimated by studying the intra-assay and inter-assay variations. Serum pools of very low, low, medium, high-medium, and high PSL concentrations were utilized for determining the level of imprecision in the assay by assessing the PSL in each pool eight times in the assay and in eight different assays. **Table 6** depicts the intra and inter-assay coefficient of variations. The coefficient of variation (CVs) of five serum pools for intra and inter-assay variation (n=8 and N=8, replicate of each pool) was< 8.46%. The percentage of coefficient of variation is calculated as % CV = (SD/X) * 100 at a particular analyte level, where X = mean, SD = standard deviation, and CV = coefficient of variation.

**Table 6:** Inter and intra-assay coefficient of variation for the measurement of prednisolone in human serum pools using the PSL-21-HS-BSA-antibody and PSL-21-HS-U-HRP-enzyme conjugate.

Variation	Sample value (ng/mL) (mean ± S.D.)	Coefficient of variation (%)
Intra-assay *n* = 8	0.80 ± 0.02 1.41± 0.10 5.45 ± 0.21 15.72 ± 0.84 25.85 ± 0.94	2.50 7.09 3.85 5.34 3.63
Inter-assay *N*= 8	0.77 ± 0. 05 1.30 ± 0.11 14.92 ± 0.22 15.72 ± 0.44 26.02 ± 1.04	6.49 8.46 1.47 2.79 3.99

The intra-assay coefficients of variation ranged from 2.50%–7.09% whereas, the inter-assay coefficients of variation ranged from 1.47%– 8.46%.

### 4.8 Correlation coefficient

**Figure 4:**
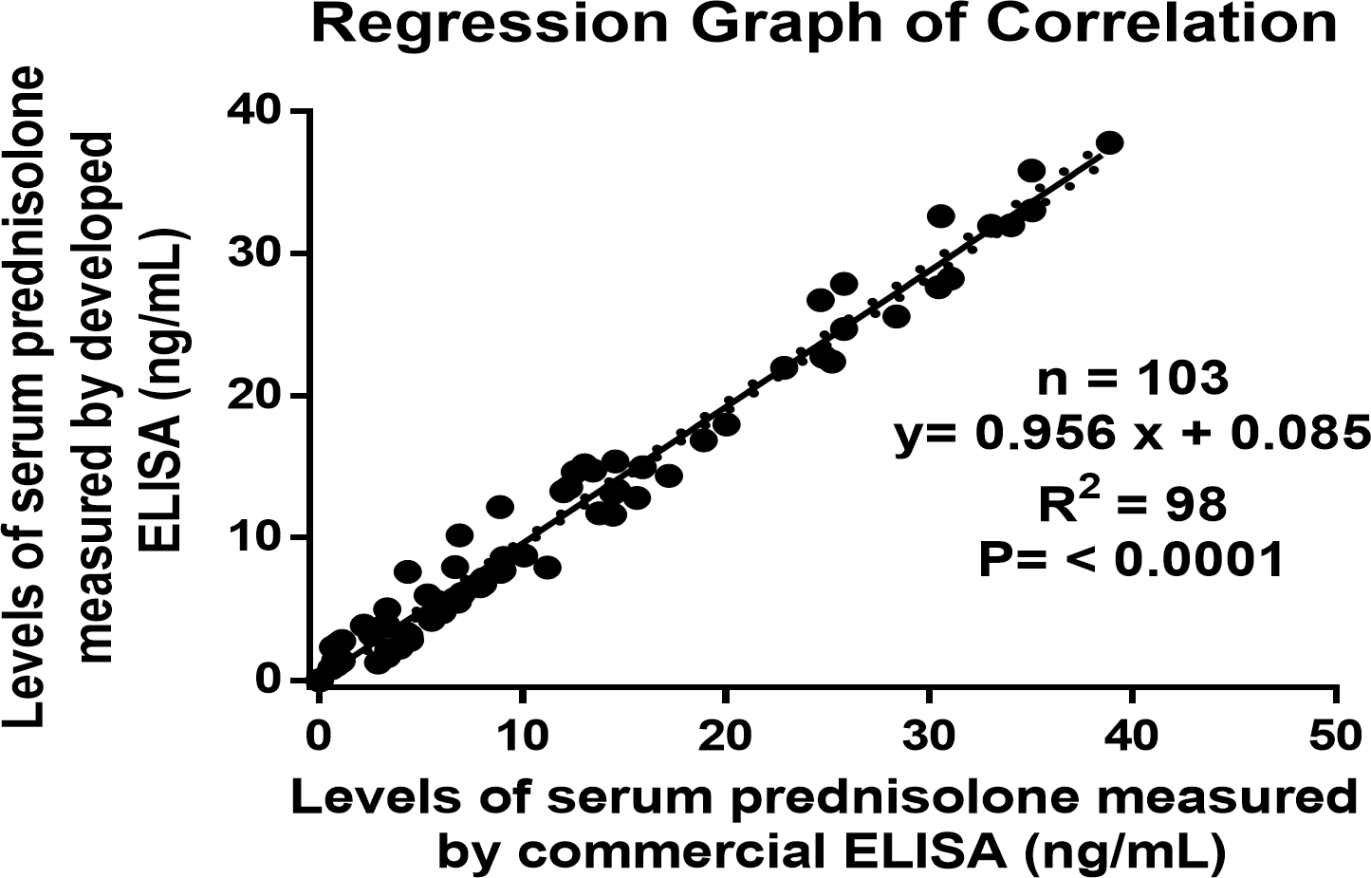
Regression graph of the correlation between the serums prednisolone concentrations as estimated by the developed ELISA and an established ELISA kit.

The newly developed technique must yield results analogous to those of the already available conventional method. The correlation coefficient for values of PSL in serum samples (n = 103) measured by developed ELISA and ELISA kit of prednisolone purchased from Sincere Biotech Co. Ltd., Beijing, China was found to be r^2 ^= 0.98. The regression analysis was performed in which both X and Y were subject to measurement error. **Figure 4** shows that the method fit a straight line to a two-dimensional data where both the variables, X and Y, are measured with errors that can accommodate differences in measurement error between the test and the reference method. The linear regression curve of the correlated data was plotted using Graph Pad Prism version 6.0 for Microsoft Windows (42–46).

### 4.9 Estimation of prednisolone in patients treated for different diseases

**Figure 5:**
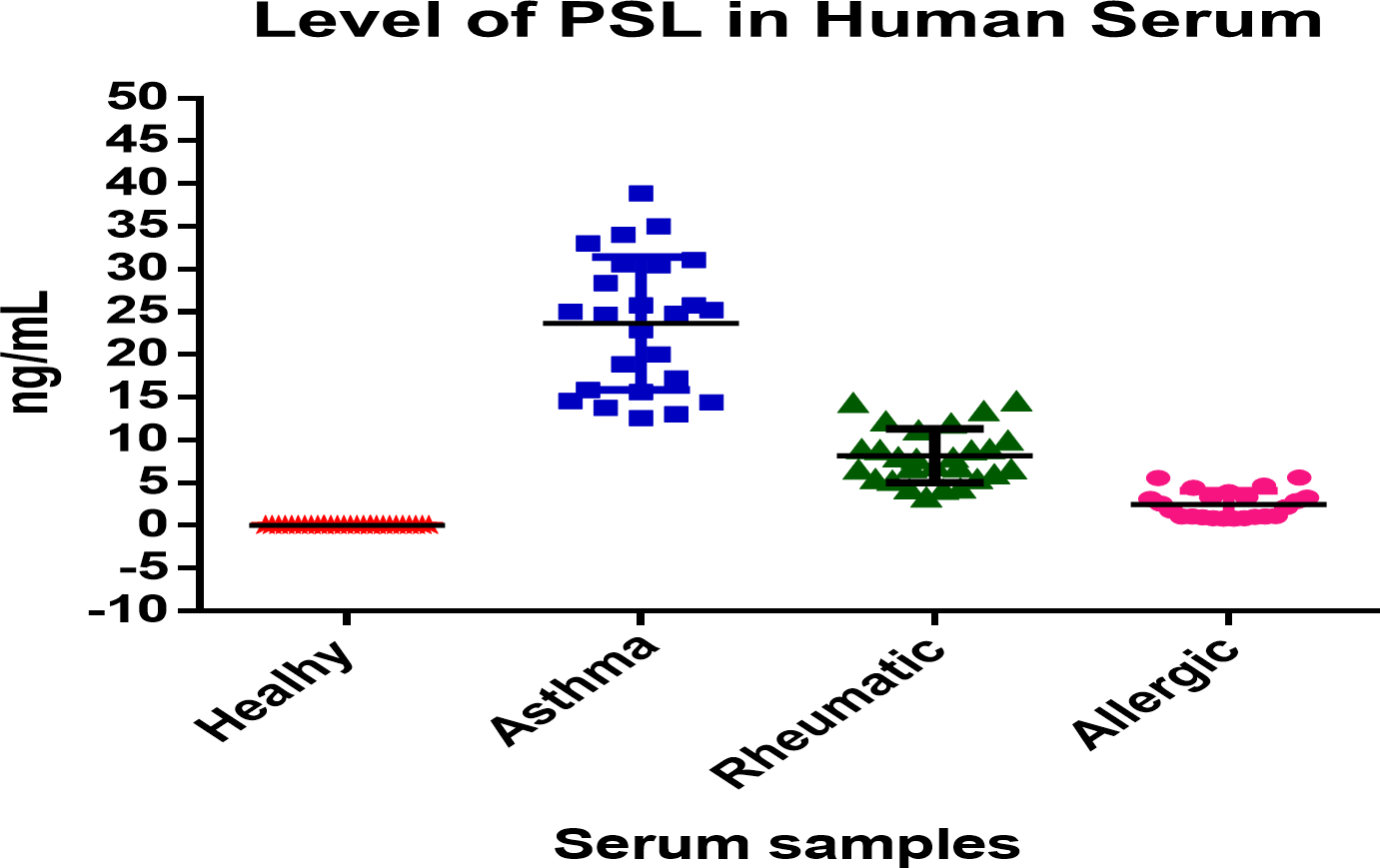
Box-and-Whisker diagram of level of prednisolone in human serum samples estimated by developed ELISA.

We determined the prednisolone concentration in human serum using samples from healthy volunteers (17 males and 11 females) and in human patients taking treatment with prednisolone through oral, local, or injection forms for chronic asthma (20 males and 5 females), rheumatoid arthritis (16 males and 11 females), and allergies (11 males and 12 females) with ages between 10 to 72 years. We followed all institutional guidelines and ethical approval as per the norm was taken before the initiation of the study (44–46). Samples values as determined by the developed ELISA are described in **Table 7** and also presented in the form of a box and whisker diagram (plotted using Graph Pad Prism version 6.0 for Microsoft Windows), as shown in **Figure 5**.

**Table 7:** Estimation of prednisolone by developed ELISA in human serum of healthy volunteers and patients taking treatment.

Variable	Healthy		Asthma		Rheumatic		Allergic	
Gender	Male	Female	Male	Female	Male	Female	Male	Female
No. of samples	17	11	20	5	16	11	11	12
Age	18-64		10 -72		40-62		16-60	
Prednisolone (mean ± SD) (ng/mL)	(0.00 ± 0.00)		(24.06 ± 8.09)		(8.17 ± 3.15)		(1.80 ± 1.24)	

## 5 Discussion

In the present study, we investigated the use of different spacers in enzyme conjugates to develop a sensitive and specific enzyme-linked immunosorbent assay (ELISA) for prednisolone. Four homobifunctional spacers, namely ADH, CH, EDA, and urea, were introduced between the PSL-21-HS carboxyl derivative of prednisolone and horseradish peroxidase (HRP) as the enzyme label. The influence of spacer length on the functional parameters of the prednisolone ELISA was examined. The terminal functional groups of these spacers do not spontaneously react with the functional group of steroid/carrier protein/enzyme, and therefore, necessitate some bioconjugate reagent as an in-between to carry out the effective coupling of these spacers (10). Carbodiimide along with N-hydroxysuccinimide has been the optimal reagent for coupling steroid and proteins through spacers as they do not introduce any additional atoms in the conjugate.

The data of the present investigation revealed that the insertion of spacers in the enzyme conjugate affected the lower detection limit and ED50 of the PSL assay. The assays using ADH, CH, EDA, and urea spacers exhibited sensitivities of 1.22 ng/mL, 0.59 ng/mL, 0.48 ng/mL, and 0.018 ng/mL, respectively. Among the different combinations, the PSL-21-HS-BSA antibody and PSL-21-HS-urea-HRP enzyme conjugate demonstrated the highest sensitivity and ED_50_.

The rigid nature of the urea spacer, with its atomic length of three and non-aliphatic, hydrophilic properties, may have contributed to the enhanced sensitivity of the assay. This rigidity could have created a greater distance between the prednisolone moiety and the enzyme, reducing steric hindrance and allowing easier access for unlabeled prednisolone to bind to the antibody. The findings suggest that the nature of the spacer, rather than its length, is more relevant to assay sensitivity. Previous studies have also reported similar observations, highlighting the potential of hydrophilic and rigid spacers to improve assay sensitivity and specificity. It has also been previously reported that the spacer length does not bear any correlation with the assay sensitivity in ELISA (1–5, 11, 19, 20) as it does have an effect in some other immunoassays and auxiliary binding systems (9–11, 20). The use of a hydrophilic and rigid spacer possibly helped in projecting the hapten away from the enzyme, leading to an enhanced antibody binding signal and improved sensitivity of the assay (9–11, 14, 20).

Since the assay was based on superior sensitivity, ED50, affinity, and specificity, the combination of PSL-21-HS-BSA antibody and PSL-21-HS-urea-HRP enzyme conjugate was selected for further analysis. The recovery of PSL from exogenously spiked human serum pools ranged from 88.32% to 102.50%, and the intra-assay and inter-assay coefficients of variation were<8.46%. Additionally, the correlation coefficient between the developed ELISA and a commercially available kit for serum PSL measurement was found to be r^2^ = 0.98 and the correlation coefficient for values of prednisolone in serum samples (n = 103).

The results of the present study were compared to the commercially validated accepted kit, the developed assay showed advantages such as simplicity, single-step immunoassay procedure, fewer washing steps, lower intra-assay and inter-assay variability, and the provision of recovery and cross-reactivity data.

To the best of our knowledge, this study is the first to compare the impact of different spacers in enzyme conjugates on the analytical variables of an ELISA for prednisolone. All the reagents used in the assay, including buffers, immunogen, antigen-enzyme conjugate, and antibody generation, were developed in-house.

## 6 Conclusions

In the present study, the introduction of different spacers in enzyme conjugates affected the sensitivity and specificity of the prednisolone ELISA. The rigid nature of the urea spacer demonstrated improved sensitivity and reduced steric hindrance. Due to this rigid nature of urea, it might have projected PSL moiety away from the enzyme which might have aided in keeping the bound antibody away from the enzyme, thereby reducing the inclusive attraction between enzyme and antibody simultaneously. The results highlight the importance of spacer properties in influencing assay performance and provide insights for the development of more sensitive and specific assays for prednisolone detection.

## Data availability statement

The original contributions presented in the study are included in the article/Supplementary Material. Further inquiries can be directed to the corresponding authors.

## Ethics statement

The experimental protocols were approved by the institutional animal ethics committee (IAEC) of the National Institute of Health and Family Welfare (NIHFW), New Delhi, India. All animal experiments were performed in accordance with the guidelines of Committee for the Purpose of Control and Supervision of Experiments on Animals (CPCSEA), Government of India. Written informed consent was obtained from the owners for the participation of their animals in this study.

## Author contributions

DK, TS, HS, and SS conceived and designed the experiments; DK, SS, HO, MK, and PB carried out the experiments; DK, HS, TS, SS, and PB analyzed the data; DS, HS, BK, SS, and PB wrote and finalized the manuscript. All authors contributed to the article and approved the submitted version.
